# PLOD2 regulated by transcription factor FOXA1 promotes metastasis in NSCLC

**DOI:** 10.1038/cddis.2017.553

**Published:** 2017-10-26

**Authors:** Hongzhi Du, Yulong Chen, Xiaoying Hou, Yue Huang, Xiaohui Wei, Xiaowen Yu, Shuyun Feng, Yao Wu, Meixiao Zhan, Xin Shi, Sensen Lin, Ligong Lu, Shengtao Yuan, Li Sun

**Affiliations:** 1 Jiangsu Key Laboratory of Drug Screening, China Pharmaceutical University, Nanjing, China; 2 Department of Thoracic/Head and Neck Medical Oncology, University of Texas MD Anderson Cancer Center, Houston, TX, USA; 3 Interventional Radiology Center, Zhuhai Precision Medicine Center, Zhuhai People's Hospital of Tongji University, Zhuhai, China; 4 Department of General Surgery, Southeast University Affiliated Zhongda Hospital, Nanjing, China; 5 Jiangsu Center for Pharmacodynamics Research and Evaluation, China Pharmaceutical University, Nanjing, China

## Abstract

In multiple types of tumors, fibrotic collagen is regarded as the 'highway' for cancer cell migration, which is mainly modified by lysyl hydroxylase 2 (PLOD2). The previous findings have demonstrated that the expression of PLOD2 was regulated by multiple factors, including HIF-1*α*, TGF-*β* and microRNA-26a/b. Although PLOD2 was confirmed to be related to poor prognosis in lung adenocarcinoma, the regulatory mechanism and function of PLOD2 in human lung adenocarcinoma is poorly understood. On the other hand, upregulation or hyperactivation of epidermal growth factor receptor is considered as a prognostic marker in many cancers, especially in non-small-cell lung cancer (NSCLC). In this study, we found that PLOD2 was elevated in NSCLC specimens and positively links to NSCLC poor prognosis. Gain- and loss-of-function studies and orthotopic implantation metastasis model pinpointed that PLOD2 promotes NSCLC metastasis directly by enhancing migration and indirectly by inducing collagen reorganization. In addition, we revealed that PLOD2 was regulated by PI3K/AKT-FOXA1 axis. The transcription factor FOXA1 directly bound to the PLOD2 promoter, and turned on PLOD2 transcription. In summary, our findings revealed a regulatory mechanism of NSCLC metastasis through EGFR-PI3K/AKT-FOXA1-PLOD2 pathway, and provided PLOD2 as a therapeutic target for NSCLC treatment.

Nowadays, the survival of patients with malignant tumors has improved owing to the development of advanced treatment options. Unfortunately, tumor metastasis remains one of the main causes of death among malignant tumor patients.^1^ Previous studies regarding tumor metastasis have primarily focused on the adhesion and migration ability of cancer cells themselves. However, mounting evidences suggest that tumorigenesis and progression are determined not only by tumor cells but also by the tumor microenvironment (TME),^2, 3, 4^ which support the 'seed [tumor cells] and soil [tumor stroma]' hypothesis. Moreover, the extracellular matrix (ECM), as the chief component of the TME, has important roles in multiple stages during tumor progression, including adhesion, migration, proliferation, differentiation and survival, especially in tumor metastasis.^5^ Recently, many therapeutic strategies have been designed to target both TME and tumor cells.^6, 7, 8^

Furthermore, collagens, the most abundant proteins that provide the scaffold for ECM assembly, seem to be the 'highway' for cancer cell migration.^9, 10, 11^ Increasing evidence has shown that collagens not only provide a barrier for migration but also promote metastasis based on different collagen organizations. Results from multiple types of human cancers suggest that the accumulation of stabilized collagen is enhanced by different covalent intra- and intermolecular crosslinks.^10, 11, 12, 13^ The different types of collagen organization are determined after crosslink formation by the hydroxylation of collagen telopeptidyl and helical Lys residues.^13, 14^ These changes are primarily mediated by lysyl hydroxylases 2, which is encoded by distinct procollagen-lysine, 2-oxoglutarate 5-dioxygenase 2 (*PLOD2*) gene, while lysyl oxidase (LOX) also mediates the stabilized collagen crosslink after hydroxylation by PLOD2.^10, 15, 16^ PLOD2 has recently confirmed to be related to poor prognoses in breast cancer,^15^ hepatocellular carcinoma,^17^ pancreatic cancer,^18^ sarcomas,^10^ renal cell carcinoma^19^ and lung adenocarcinoma.^13^ It has been reported that the expression of PLOD2 is regulated by HIF-1*α* in sarcomas,^10^ pancreatic cancer^18^ and breast cancer,^15^ inhibited by microRNA-26a/b in renal cell carcinoma,^19^ whereas induced by TGF-*β* in myofibroblasts.^20^ Although the expression of PLOD2 was modulated by multiple factors, the regulation of PLOD2 in lung cancer is still unknown.

Although PLOD2 is believed to negatively correlate to poor prognosis in murine lung adenocarcinoma,^13^ the regulation mechanism and function of PLOD2 in human lung adenocarcinoma is poorly understood. Clinically, EGFR is a prognostic marker and an effective therapeutic target of multiple human cancers, especially NSCLC.^21^ However, it is well known that the EGFR inhibitors widely face primary resistance (~60%) and rapidly generate acquired resistance (6–12 months).^22, 23^ Therefore, the downstream effector function of EGFR may act as a substitutable therapeutic target of EGFR to overcome the drug resistance.^24, 25^ The previous study^26^ showed that EGFR inhibition attenuated liver fibrosis and the development of hepatocellular carcinoma. Given that PLOD2 is a key enzyme accounting for fibrosis, it is interesting to examine whether EGFR regulates fibrosis through PLOD2.

In this study, we demonstrated that PLOD2 could be regulated via the PI3K/AKT signaling pathway driven by EGFR *in vitro* and *in vivo*. Meanwhile, we also confirmed that PLOD2 promotes NSCLC metastasis directly by enhancing migration and indirectly by inducing collagen reorganization. Furthermore, the transcription factor FOXA1, downstream of PI3K/AKT, directly turned on the transcription of *PLOD2* gene. Collectively, PLOD2 can be regulated by FOXA1 via the PI3K/AKT signaling pathway, which can be activated by several stimuli (EGFR, TGF-*β*, HIF-1*α*, VEGFR etc.). Our results suggest that PLOD2 is a vital factor for NSCLC progression, and provide PLOD2 as a potential target for NSCLC treatment.

## Results

### PLOD2 closely relates to the phosphorylation of EGFR

**Figure 1:**
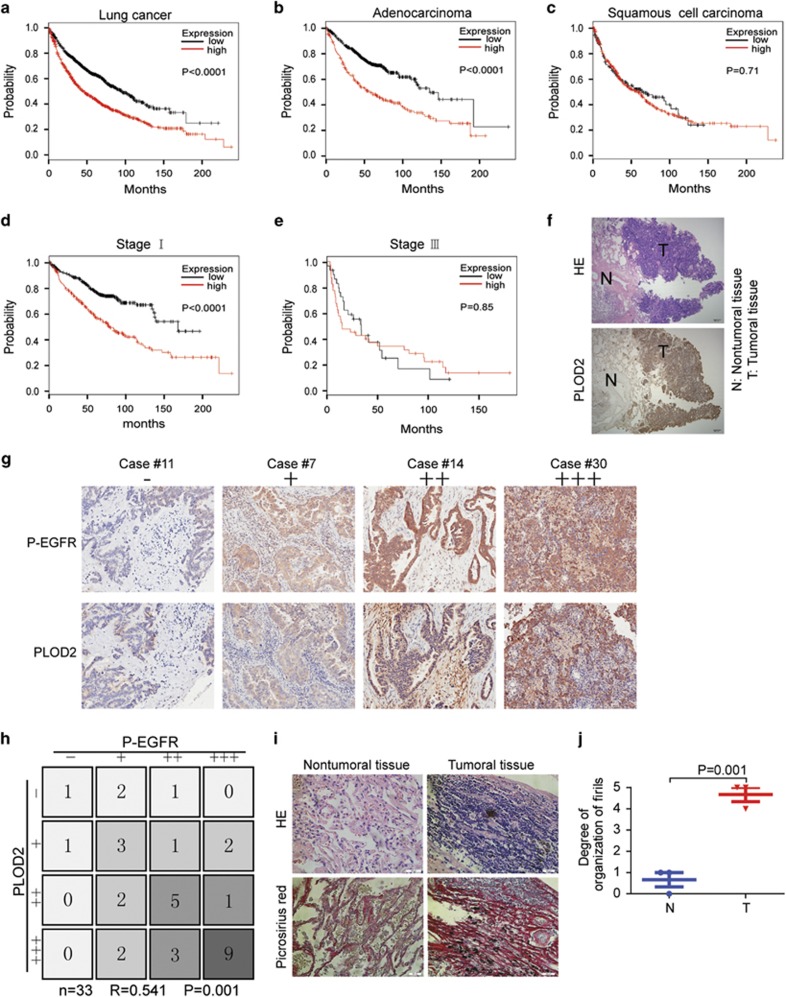
PLOD2 is closely related to the P-EGFR (epidermal growth factor receptor). (**a**–**e**) Kaplan–Meier (KM) analysis of relapse-free survival in lung cancer patients was from KM plotter (http://kmplot.com/analysis). PLOD2 in lung cancer patients (*n*=1926, **a**), in lung adenocarcinoma patients (*n*=719, **b**), in lung squamous cell carcinoma patients (*n*=525, **c**), in lung adenocarcinoma patients at stage I (*n*=578, **d**) and in lung adenocarcinoma patients at stage III (*n*=70, **e**). (**f**) The high expression of PLOD2 in tumor tissue of clinical sample by immunohistochemistry (IHC). (**g** and **h**) The expression levels of P-EGFR and PLOD2 were consistently analyzed in 33 human lung adenocarcinoma specimens. (**i** and **j**) The fibrillate collagen formation and collagen deposition were detected by hematoxylin and eosin (HE) staining and picrosirius red staining in clinical samples

To explore the correlation of PLOD2 with lung cancer prognosis, the relation was analyzed by the Kaplan–Meier plotter (http://kmplot.com/analysis). The data suggested that PLOD2 was associated with poor prognosis of lung cancer patients (Figure 1a), especially in lung adenocarcinoma patients (Figure 1b) but not in lung squamous cell carcinoma patients (Figure 1c). Further analysis showed that PLOD2 was an adverse prognosis marker in lung adenocarcinoma patients at stage I but not at stage III (Figures 1d and e) and was independent of smoking history and gender (Supplementary Figures S1a and b). Meanwhile, the clinical sample analysis also showed that the expression of PLOD2 was higher in tumor tissue than in normal tissue (Figure 1f). It is well known that phosphorylation on EGFR is a key event for EGFR pathway activation. The preliminary results revealed that PLOD2 might be closely related to the phosphorylation of EGFR (P-EGFR) in NSCLC cell lines (Supplementary Figures S1c–e). The expression levels of both P-EGFR and PLOD2 were higher in adenocarcinoma tissues than that in normal tissues (Supplementary Figure S1f). Moreover, the coexpression analysis suggested that P-EGFR and PLOD2 were simultaneously expressed in adenocarcinoma tissues and tissue microarray (Supplementary Figures S1g and 1h), which were consistent with the results shown in NSCLC cell lines (Supplementary Figures S1c–e). PLOD2 is a significant collagen synthetase that can induce collagen reorganization, providing a stabilized 'highway' for cancer cell migration.^10^ Therefore, collagen deposition and fibril organization were detected by picrosirius red staining, which showed that collagen deposition was increased and the degree of fibrillar organization was enhanced in human lung adenocarcinoma tissues (Figures 1i and j). These staining results suggested that modifying enzymes such as hydroxylases and LOX can be enhanced by some oncogenes in adenocarcinoma tissues. In sum, the relationship between P-EGFR and PLOD2 indicated that some regulations possibly exist.

### EGFR inhibitor decreases NSCLC metastasis via PLOD2 *in vitro* and *in vivo*

Given that our above results showed the relationship between P-EGFR and PLOD2, it was worthy to test whether P-EGFR controls PLOD2 level. Interestingly, the EGFR inhibitor Gefinitib treatment induced time- and dose-dependent PLOD2 decrease in NCI-H1975 and HCC827 cells (Supplementary Figures S2a–h), which are EGFR-mutant NSCLC cell lines. Additionally, the recognized EGFR-mutant lung cancer cell line, NCI-H1975, conferred resistance to first-generation EGFR TKIs with mutation of T790M. However, WZ4002, the novel mutant-selective EGFR kinase inhibitor against EGFR T790M, also significantly inhibited the expression of PLOD2 at extremely low concentration (Supplementary Figures S2a and 3a).

**Figure 2:**
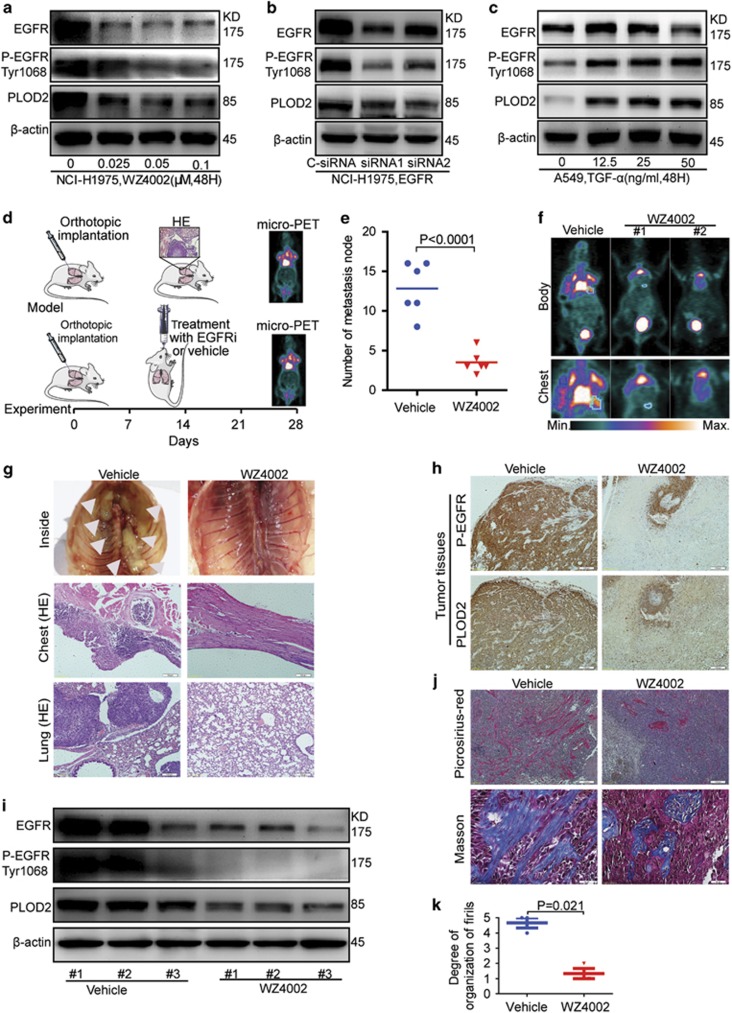
Epidermal growth factor receptor (EGFR) inhibitor decreased metastasis possibly via PLOD2, *in vitro* and *in vivo*. (**a**) WZ4002 inhibited the expression of PLOD2 in the NCI-H1975 cell line. (**b**) Knockdown of EGFR decreased the expression of PLOD2 by small interfering RNA (siRNA) in the NCI-H1975 cell line. (**c**) The EGFR ligand (tumor growth factor-*α* (TGF-*α*)) promoted the expression of PLOD2 in the A549 cell line. (**d**) The protocol of the NSCLC metastasis model generated by orthotopic implantation. (**e**) The number of metastasis nodes was decreased by WZ4002. (**f**) Representative micro-positron emission tomography (micro-PET) image of mice 4 weeks after implantation. The color scale is indicated. (**g**) Further confirmation of the metastasis nodes on the chests and lungs by hematoxylin and eosin (HE) staining. (**h** and **i**) The expression of P-EGFR and PLOD2 was consistently decreased by WZ4002 in the tumor tissues. (**j** and **k**) Fibrillar collagen formation and collagen deposition detected by picrosirius red and Masson’s trichrome staining in the tumor tissues.The degree of fibrillar organization was evaluated by a pathologist. The score (from 0 to 5) indicates the degree of fibrillar organization. Higher score suggested greater fibrillar organization. The data were represented as the mean±S.D. of three independent experiments. The *P*-values <0.05 were considered statistically significant for all tests

Either EGFR silence or another EGFR inhibitor (Erlotinib) treatment substantially downregulated the levels of PLOD2 in NCI-H1975 and HCC827 cells (Figure 2b and Supplementary Figures S3b–e). Conversely, the EGFR ligands EGF and TGF-*α* treatment induced PLOD2 in EGFR-responsive NSCLC A549 cells (Figure 2c and Supplementary Figure S3f). Taken together, our findings showed that EGFR is an important regulator for PLOD2 expression.

**Figure 3:**
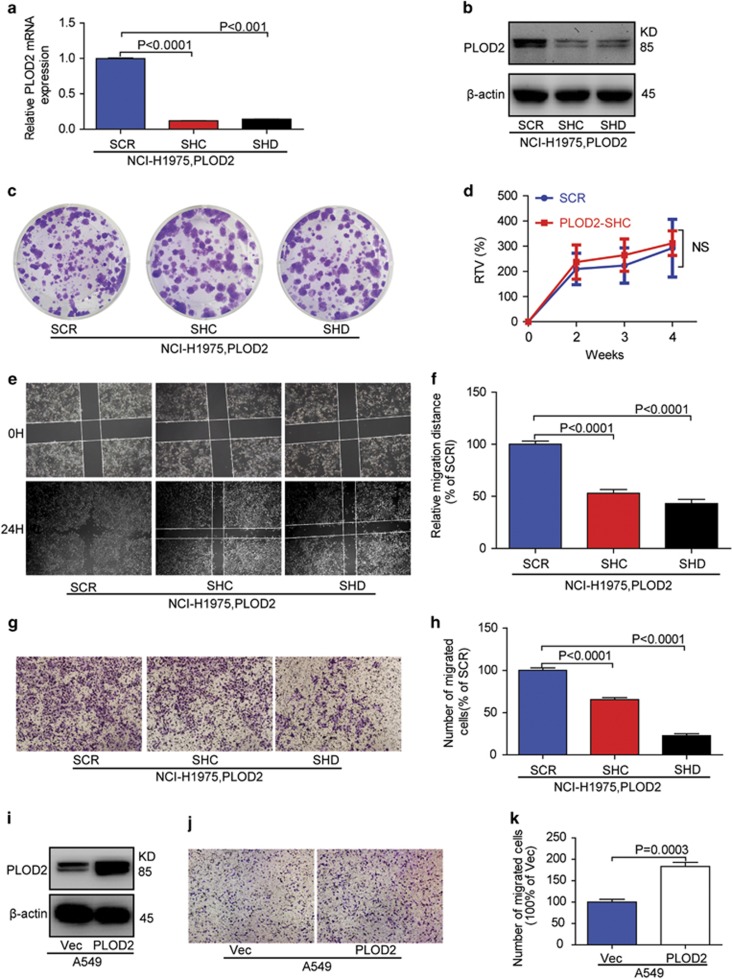
PLOD2 increased the migration of the cancer cells. (**a** and **b**) The expression of PLOD2 was knocked down by short hairpin RNA (shRNA) in the NCI-H1975 cell line. (**c**) The proliferation of PLOD2-knockdown cells was evaluated by clone formation. (**d**) The proliferation of PLOD2-knockdown cells was evaluated in a subcutaneous transplant mouse model. (**e** and **f**) The migration of PLOD2-knockdown cells was evaluated by a wound-healing assay. (**g** and **h**) The migration of PLOD2-knockdown cells was evaluated by transwell chambers. (**i**–**k**) The migration of PLOD2-overexpressing cells was evaluated by transwell chambers

Since our above results showed that EGFR activated PLOD2 expression, we next wanted to examine the effect of PLOD2 in EGFR-mediated oncogenic function. In the metastasis model of orthotopic implantation (Figure 2d), the EGFR inhibitor not only decreased the number of metastasis nodes but also improved the quality of life, which was monitored by body weight (Figure 2e and Supplementary Figure S3g). Consistent with the previous data, the chest walls were diffusely covered with tumor nodes in the mode group,^27, 28^ whereas metastasis nodes were markedly decreased in the inhibitor group. This result was also validated by micro-PET via ^18^F-DG and HE (Figures 2f–g). Furthermore, the expression of PLOD2 was inhibited along with the inhibition of P-EGFR by WZ4002 *in vivo* (Figures 2h–i). Furthermore, the picrosirius red staining and Masson’s trichrome of tumors showed that the decrease in metastasis was possibly due to PLOD2-induced collagen deposition and fibrillar organization (Figures 3j–k), consistent with the previous report.^10^ Collectively, our results inferred that EGFR inhibitors attenuated NSCLC metastasis, at least in part through hampering the expression of PLOD2.

### PLOD2 promotes the NSCLC metastasis *in vitro* and *in vivo*

Next, we needed to study the role of PLOD2 in lung cancer progression. Surprisingly, deletion of PLOD2 did not show any detectable effect on cell proliferation *in vitro* and *in vivo* (Figures 3a–d). However, knockdown of PLOD2 blocked the migration in NCI-H1975 cells (Figures 3e–h), whereas overexpression of PLOD2 played an opposite role in the A549 cells (Figures 3i–k). In addition, Minoxidil, a PLOD2 inhibitor, could also inhibit NCI-H1975 cell migration (Supplementary Figures S4a–d). These results indicate that PLOD2 was a positive regulator for NSCLC cell metastasis.

To address whether PLOD2 localizes downstream of EGFR pathway to modulate NSCLC migration, we carried out epistasis assay. Knockdown of PLOD2 partially but effectively reversed A549 cell migration induced by TGF-*α* stimulation (Supplementary Figures S4e–h). Additionally, overexpression of PLOD2 could partially neutralize the effect upon cell migration of the EGFR inhibitor (WZ4002) in NCI-H1975 cells (Supplementary Figures S4i–l). Therefore, these results together with our existing data confirmed that PLOD2 was one target protein of the EGFR signaling pathway for migration.

**Figure 4:**
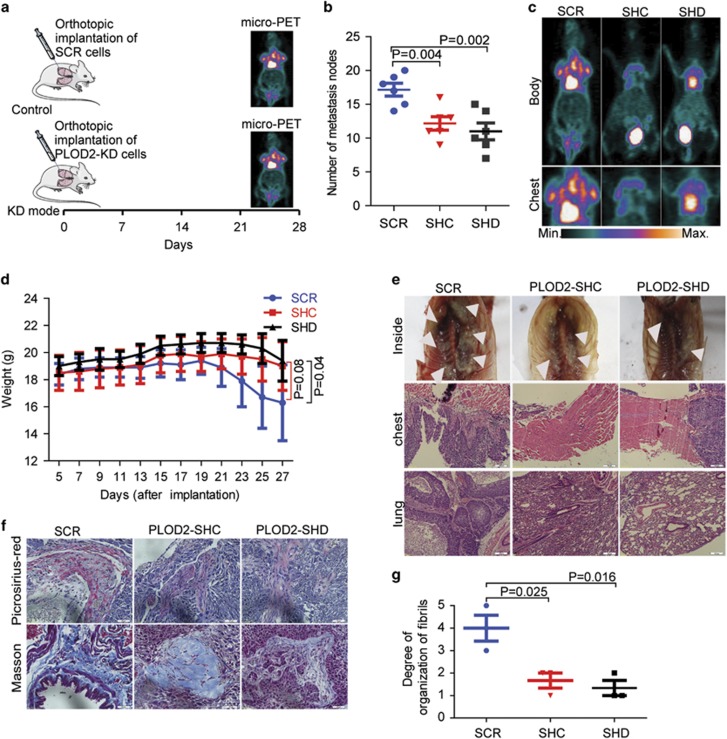
Knockdown of PLOD2 decreased the metastasis *in vivo*. (**a**) The protocol of the development of the NSCLC metastasis model based on orthotopic implantation. (**b**) Knockdown of PLOD2 decreased metastasis *in vivo*. (**c**) Representative micro-positron emission tomography (micro-PET) image of mouse, 4 weeks after implantation. The color scale is indicated. (**d**) Knockdown of PLOD2 improved the life quality of the NSCLC metastasis model. (**e**) The metastasis nodes were outside and inside the chest based on gross examination. The metastasis nodes on the chests and the lungs were further confirmed by hematoxylin and eosin (HE) staining. (**f** and **g**) Fibrillar collagen formation and collagen deposition was detected by picrosirius red and Masson’s trichrome staining in the tumor tissues, when PLOD2 was knocked down by short hairpin RNA (shRNA). The degree of fibrillar organization was evaluated by a pathologist. The score (from 0 to 5) indicates the degree of fibrillar organization. Higher score suggested greater fibrillar organization. The data were represented as the mean±S.D. of three independent experiments. The *P*-values <0.05 were considered statistically significant for all tests

In the metastasis model of orthotopic implantation (Figure 4a), knockdown of PLOD2 decreased the number of metastasis nodes and improved the quality of life (Figures 4b and d). After 4 weeks, compared with the control groups, metastasis nodes were markedly decreased in chest walls when PLOD2 was silenced. The effect was also confirmed by micro-PET (Figure 4c) via ^18^F-DG and HE staining (Figure 4e). Consistent with these findings, picrosirius red and Masson’s trichrome staining of tumors showed that the aligned collagen in the PLOD2-knockdown tumor tissues was decreased compared with the control group, suggesting that the aligned 'highway' for cancer cell migration reversed (Figures 4f–g). Thus, PLOD2 induced a collagen reorganization in NSCLC cells to promote metastasis, as is observed in breast cancer^15^ and sarcomas.^10^ In conclusion, PLOD2 promoted NSCLC metastasis directly by enhancing migration and indirectly by inducing collagen reorganization.

### PLOD2 is regulated by the PI3K/AKT signaling pathway

**Figure 5:**
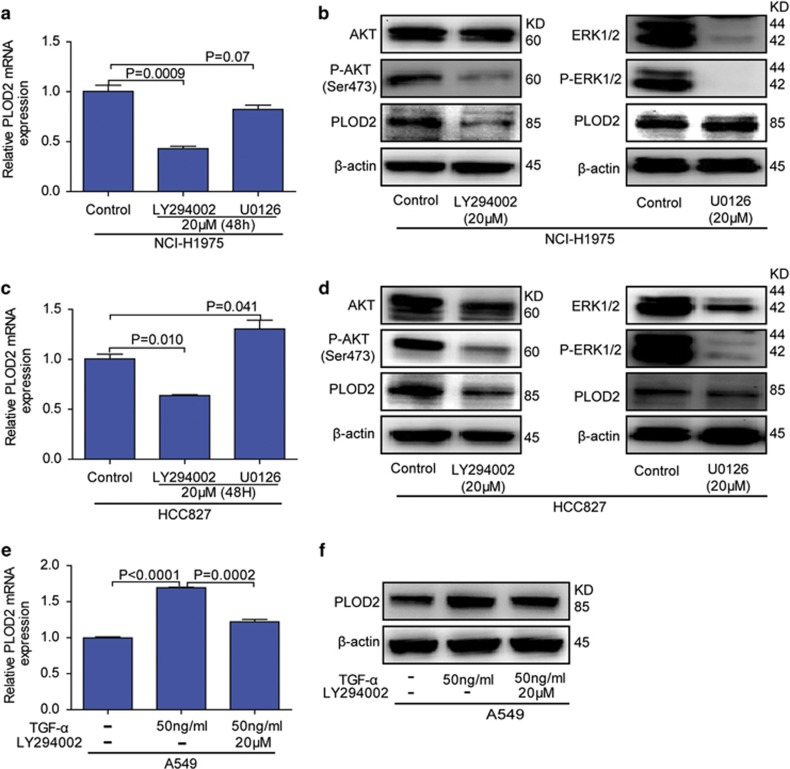
The P-EGFR (epidermal growth factor receptor) regulated the expression of PLOD2 via the phosphatidylinositol-3-kinase/AKT (PI3K/AKT) signaling pathway. (**a** and **b**) The effect of the PI3K inhibitor (LY294002) and the MEK inhibitor (U0126) on the expression of PLOD2 was detected in the NCI-H1975 cell line. (**c** and **d**) The effect of the PI3K inhibitor (LY294002) and the MEK inhibitor (U0126) on the expression of PLOD2 was detected in the HCC827 cell line. (**e** and **f**) The effects of the PI3K inhibitor (LY294002) and the EGFR ligand (tumor growth factor-*α* (TGF-*α*)) were detected in A549 cell line. The data were represented as the mean±S.D. of three independent experiments. The *P*-values <0.05 were considered statistically significant for all tests

Evidence has suggested that the PI3K/AKT and MEK/ERK pathways are the most significant classic EGFR signaling pathways that can also be regulated by other stimulus (TGF-*β*, HIF-1*α*, VEGFR etc.). Therefore, it should address that EGFR regulates PLOD2 through PI3K/AKT or MEK/ERK. The expression of PLOD2 was exclusively decreased by PI3K inhibitor LY294002 in NCI-H1975 cells (Figure 5a) but not by MEK inhibitor U0126 (Figure 5b), suggesting that PLOD2 expression was regulated by the PI3K/AKT pathway. We confirmed these results in another NSCLC cell line, HCC827 (Figures 5c–d). Furthermore, in A549 cells, TGF-*α* sustainably elevated the expression of PLOD2, which was neutralized by PI3K inhibitor LY294002 (Figures 5e–f). Taken together, our findings revealed that the expression of PLOD2 is turned on through PI3K/AKT pathway in NSCLC cells.

### The PI3K/AKT signaling pathway promotes the expression of PLOD2 via FOXA1 transcription factor

**Figure 6:**
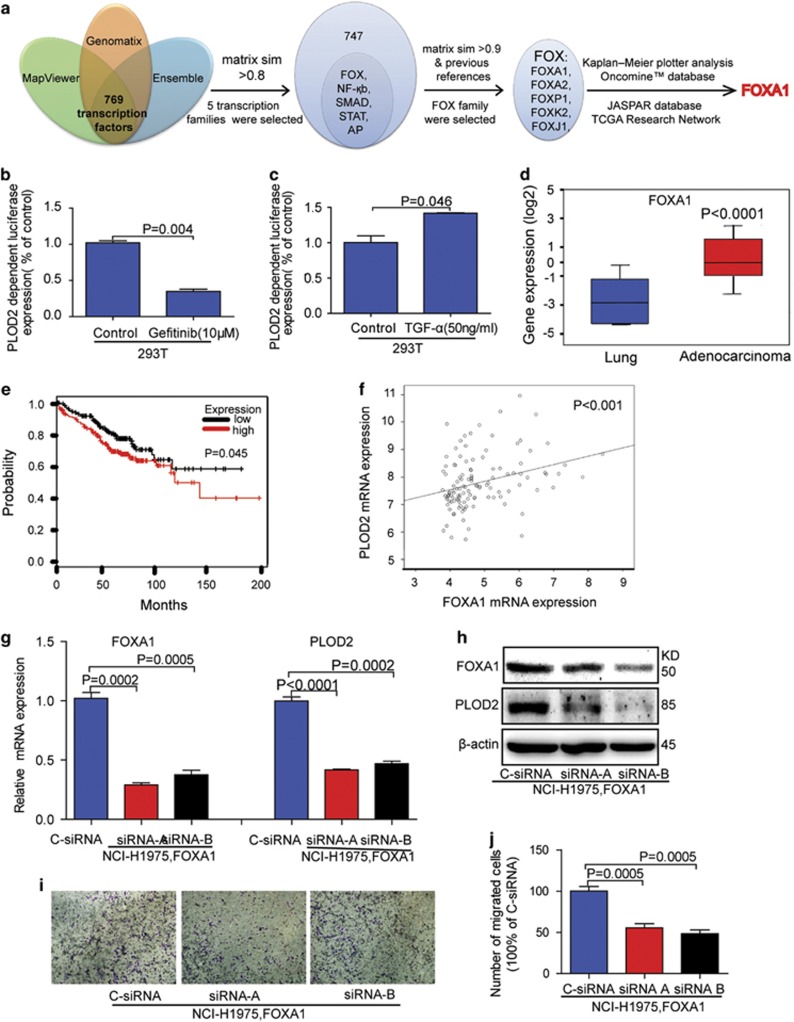
FOXA1 might be the transcription factors of PLOD2. (**a**) FOXA1 was selected as the potential transcription factor of PLOD2 based on a series of databases. First, the promoter sequence of PLOD2 was obtained via the Ensemble project and Mapviewer. Then, the 769 potential transcription factors were predicted using the Genomatix database. Finally, the five most potential transcription families were screened out based on the prediction score (the matrix sim). Based on a Kaplan–Meier (KM) plotter analysis, the Oncomine database, JASPAR database and TCGA Research Network, FOXA1 may be a potential transcription factor. (**b** and **c**) The effect of Gefinitib and tumor growth factor-*α* (TGF-*α*) on the transcription of PLOD2 was evaluated by luciferase reporter assays. (**d**) The expression of FOXA1 was higher in adenocarcinoma tissues than in normal tissues according to the Oncomine database. (**e**) KM analysis of relapse-free survival in lung cancer patients was from KM plotter. PLOD2 in lung adenocarcinoma patients at stage I (*n*=370). (**f**) Correlation analysis of FOXA1 and PLOD2 in lung adenocarcinoma patients based on the TCGA Research Network. (**g** and **h**). Knockdown of FOXA1 decreased the expression of PLOD2 by small interfering RNA (siRNA) in the NCI-H1975 cell line. (**i** and **j**) Knockdown of FOXA1 decreased the cell migration. The data were represented as the mean±S.D. The *P*-values<0.05 were considered statistically significant for all tests

Although our above studies clearly showed that PI3K/AKT pathway increased PLOD2 expression, we should explore if it upregulates PLOD2 by promoting PLOD2 transcription or by blocking PLOD2 degradation. We generated the PLOD2-luciferase reporter construct, which fuses the promoter region of *PLOD2* gene and firefly luciferase sequence. Through dual-luciferase assays we found that EGFR positively regulated the transcription of PLOD2 (Figures 6b–c). These results indicated that a missing link exists between PI3K/AKT and PLOD2 transcription. To find this link, we next sought to determine the transcription factors of PLOD2 as shown schematically in Figure 6a. The five most potential transcription families were screened out based on the prediction score (the matrix sim) as shown in Supplementary Table 1. Based on the Kaplan–Meier plotter analysis, the Oncomine database and the JASPAR database, FOXA1 may be a potential transcription factor (Figures 6d and e).The correlation analysis between FOXA1 and PLOD2 based on the TCGA Research Network also confirmed FOXA1 as the transcription factor of PLOD2 (Figure 6f). Furthermore, knockdown of FOXA1 indeed decreased PLOD2 levels in NCI-H1975 and HCC827 cells (Figures 6g–h and Supplementary Figures S5a and b). The migration of NSCLC cells was also inhibited when FOXA1 was knocked down. These data strongly suggested that PI3K/AKT increased the PLOD2 level, maybe through the transcription factor FOXA1.

**Figure 7:**
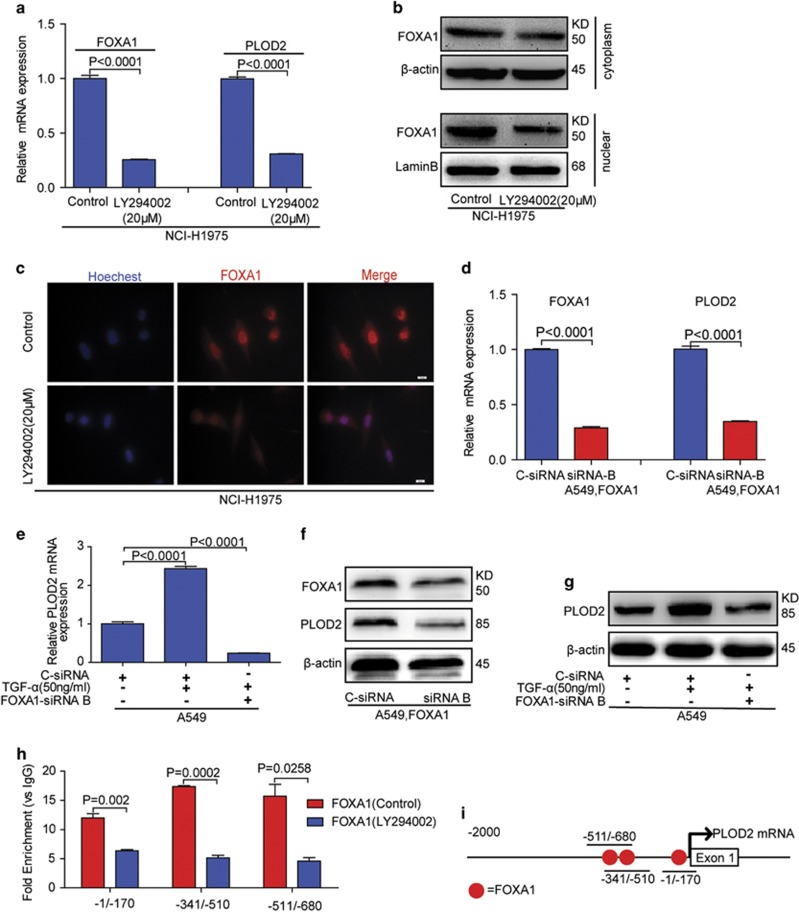
The phosphatidylinositol-3-kinase/AKT (PI3K/AKT) signaling pathway promoted the expression of PLOD2 via the FOXA1 transcription factors. (**a**) The PI3K/AKT signaling pathway inhibitor (LY294002) consistently decreased the expression of FOXA1 and PLOD2. (**b** and **c**) The PI3K/AKT signaling pathway inhibitor (LY294002) inhibited the nuclear translocation by separation of the cytoplasm and nucleus, as revealed by the immunofluorescence assay. (**d**) Knockdown of FOXA1 decreased the expression of PLOD2 by small interfering RNA (siRNA)in the A549 cell line. (**e**–**g**) Knockdown of FOXA1 reversed the upregulation of *PLOD2* induced by tumor growth factor-*α* (TGF-*α*) in A549 cell line. (**h**) FOXA1 bind to three putative bind regions upstream of the transcription start site (TSS) of *PLOD2* gene. PI3K/AKT signaling pathway inhibitor (LY294002) inhibited the bind regions of *PLOD2* gene. Precipitated DNA fragments in ChIP assays were examined by quantitative reverse transcription-PCR (qRT-PCR). Immunogobulin G (IgG) was used as a negative control. (**i**) Schematic representation of the PLOD2 locus. FOXA1-binding sites upstream of the TSS of PLOD2 were predicted by ChIP assay. The *P*-values <0.05 were considered statistically significant for all tests

**Figure 8:**
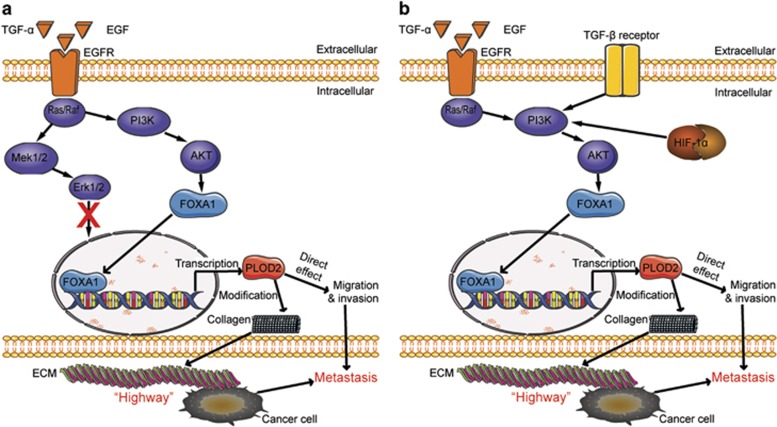
The schematic representation of the regulatory mechanism about PLOD2 was summarized. (**a**) The schematic representation of the regulatory mechanism about PLOD2 was summarized based on this study. PLOD2 can be regulated by transcription factor FOXA1 via the phosphatidylinositol-3-kinase/AKT (PI3K/AKT) signaling pathway. Meanwhile, PLOD2 promotes NSCLC metastasis directly by enhancing migration and indirectly by inducing collagen reorganization. (**b**) The schematic representation of PLOD2 is summarized based on previous reports of the regulatory mechanism and this study. Previous reports showed that PLOD2 could be regulated by tumor growth factor-*α* (TGF-*β*) and hypoxia-inducible factor-1*α* (HIF-1*α*), whereas TGF-*β* and HIF-1*α* can also activate the PI3K/AKT signaling pathway. Therefore, it is suggested that PLOD2 may be a potential therapeutic target that can be regulated by several oncogenes via PI3K/AKT signaling pathway

We next explored how PI3K/AKT pathway modulates the transcription activity of FOXA1. Blockage of PI3K/AKT pathway not only reduced the FOXA1 protein level (Figure 7a and Supplementary Figure S5c) but also inhibited FOXA1 nuclear accumulation in NCI-H1975 (Figures 7b and c) and HCC827 cells (Supplementary Figures S5d and e). In A549 cells, the increased PLOD2 induced by TGF-*α* was reversed when the FOXA1 was knocked down (Figures 7d–g), suggesting that PI3K/AKT regulated PLOD2 through FOXA1. Given that FOXA1 is a transcription factor, we needed to test whether PLOD2 was the direct target of FOXA1. The chromatin immunoprecipitation (ChIP) assays showed that FOXA1 indeed bound to the promoter region of *PLOD2* gene (Figures 7h and i). In addition, we revealed that inhibition of PI3K/AKT signaling decreased the binding between FOXA1 and PLOD2 promoter. We generated various truncated plasmids and found three putative FOXA1-binding regions in the PLOD2 promoter (Supplementary Figure S5f). The reporter assays showed that deletion of these binding regions could alleviate FOXA1-induced effect, suggesting that FOXA1 bound to the promoter of PLOD2 to activate its transcription. Taken together, our results showed that PI3K/AKT signaling upregulated PLOD2 expression by increasing FOXA1 abundance and nuclear localization, and by promoting FOXA1 binding to the PLOD2 promoter region (Figure 8a). Our studies uncovered the mechanisms by which EGFR regulated NSCLC metastasis and identified PLOD2 as an important regulator for NSCLC metastasis, paving the way for NSCLC diagnosis and clinic treatment.

## Discussion

Aligned collagen organization is regarded as the 'highway' for the migration of multiple types of cancer cells, which is mainly modified by PLOD2 via building a foundation of collagen molecular crosslinks.^29^ Several studies have demonstrated that PLOD2 is modulated by various factors, such as HIF-1-*α*, TGF-*β* and microRNA-26a/b under distinct contexts.^15, 19, 20^ Although the biological role of PLOD2 has been studied in mouse lung cancer model,^13^ whether PLOD2 is involved in human lung cancer progression is still unclear. In this study, we analyzed human lung tumor samples and found that PLOD2 was upregulated in lung cancer. Further studies showed that PLOD2 was activated by EGFR-PI3K/AKT-FOXA1 pathway. The transcription factor FOXA1 directly bound to the promoter region of *PLOD2* gene to turn on its transcription. Although EGFR has been regarded as a promising therapeutic target for NSCLC. Unfortunately, the drug resistance limits it as a wide target for NSCLC clinic treatment. Thus, there is an urgent need to find the downstream effector of EGFR to substitute the therapeutic target to overcome the drug resistance. Our results revealed that EGFR promoted NSCLC metastasis by turning on PLOD2 expression, providing PLOD2 as a substitute of EGFR for NSCLC treatment.

At first, EGFR is supposed to be the effective therapeutic target for multiple human cancers, especially in NSCLC.^30^ However, in the past decade, we have seen that EGFR inhibitor widely face primary resistance (~60%) and rapidly generate acquired resistance (6–12 months).^22, 23^ When resistance mutations of EGFR inhibitors appeared, there was a need to find new EGFR inhibitors. In fact, it usually will take over 5 years to develop one new EGFR inhibitor, which is almost impossible to satisfy the rapid acquired resistance. In this case, some effector molecules as adverse prognosis markers will also be the potential therapeutic target for cancer and even can be the reliable strategy to overcome drug resistance.^24, 25^ As shown above, PLOD2 was confirmed to be a potential therapeutic target for metastasis. Previous report^10^ also has shown that Minoxidil, an inhibitor of PLOD2, exhibits significant antimetastasis effect consistent with Supplementary Figure S4. Therefore, PLOD2 may be a potential therapeutic target inhibited by Minoxidil or other molecules, even when the patient is resistant to EGFR inhibitors. In brief, our data suggest that PLOD2 may be a potential therapeutic target independently.

Abundant evidences^31, 32, 33^ have suggested that the PI3K/AKT and MEK/ERK pathways are the most significant classical EGFR signaling pathways. Therefore, we first explored the two classical regulatory pathways shown in Figure 5. In fact, other EGFR signaling pathways such as P38 MAPK,^34^ SAPK/JNK^35^ and JAK/STAT^36^ might also be involved in the regulation of PLOD2. However, the PI3K/AKT signaling pathway, which is confirmed as the regulatory pathway of PLOD2, is not only regulated by EGFR but also by TGF-*β*, HIF-1*α*, VEGFR and so on. Therefore, the reported regulators^10, 20^ (TGF-*β* and HIF-1*α*) of PLOD2 can also depend on this pathway (summarized in Figure 8b).

Increasing evidences have suggested that FOXA1 is overexpressed or amplified in lung cancer,^37, 38^ hepatocellular carcinoma,^39^ prostate cancer,^40^ glioma^41^ and esophageal adenocarcinoma.^42^ However, FOXA1s have a different role in breast cancer, maybe owing to the different dependence of estrogen receptor (data not shown), according to Toska’s reports.^43, 44^ In NSCLC,^37^ FOXA1 promoted proliferation, invasion and migration and reduce the chemosensitivity, which was consistent with our data shown in Figure 6, supporting that FOXA1 is the transcription factor of PLOD2. Of course, the other transcription factors (NF-*κ*b, SMAD, STAT, etc.) may also be involved in the regulation of PLOD2.

This study and previously published reports^10, 13^ consistently suggest that PLOD2 promotes metastasis directly by enhancing migration and indirectly by inducing collagen reorganization. Abundant evidences^10, 18^ based on wound-healing assay and transwell assay have shown that PLOD2 promotes migration, which is consistent with our data shown in Figure 3. In addition, several researchers^10, 11^ have also found that PLOD2 can switch the collagen organization providing an aligned 'highway' for rapid migration. Furthermore, the relationship between collagen organization and the ratio between HP/LP crosslinks (HP: hydroxylysyl-pyridinoline and LP: lysyl-pyridinoline) regulated by PLOD2 was confirmed via HPLC,^45^ stiffness measurements,^13^ ultrastructural analysis,^45^ picrosirius red staining^10^ and Masson’s trichrome staining.^10, 45^ Therefore, the change in collagen organization detected by staining in tumor tissues may suggest the possibility of metastasis via the 'highway'. Likewise, aligned collagen organization is a prognostic signature for survival in human breast carcinoma,^46^ which was also confirmed in our metastasis model (Figures 2 and 4). In brief, the rapid detection of biopsies by pathological examinations (picrosirius red and Masson’s trichrome staining) may be a credible prognostic signature for clinical evaluations.

However, the mechanisms of PLOD2 in direct migration capability needs to be further explored, in consideration of the migration capability is concerned with Rho proteins such as actin, myosin and so on.^47^ Moreover, because of the significant role of collagen in metastasis, some clinical antifibrosis drugs, such as pirfenidone and silibinin, may have novel therapeutic effects for malignant tumor patients. Coincidently, we found that silibinin was under clinical trial for prostate cancer, NSCLC, hepatocellular carcinoma, radiodermatitis and drug-induced liver injury in FDA (https://clinicaltrials.gov). Our research can support the drug development of silibinin, needing to be confirmed in future experiments (data will be submitted soon). This study identified critical roles of collagen and PLOD2 in the TME. Importantly, the pathological examination of tumor collagen may be a credible prognostic signature in clinical evaluation, suggesting the possible development of therapeutic strategies targeting the TME in the future.

## Materials and methods

### Database analysis

The correlation of relapse-free survival of lung cancer patients with different gene expressions was analyzed via the Kaplan–Meier plotter (http://kmplot.com/analysis), as described previously.^48, 49^ In addition, the different gene expression in normal lung tissues and NSCLC tissues were assessed via the Oncomine database^49^ (https://www.oncomine.org). Furthermore, the correlation of two different gene expressions in NSCLC tissue was analyzed via the TCGA Research Network (http://cancergenome.nih.gov).

### Tumor tissue array

Human lung cancer specimens were collected from the National Engineering Center for Biochip at Shanghai (SBC), China. In total, 33 patients who underwent surgery for histologically proven lung adenocarcinoma were selected in this research. There were totally 15 men and 18 women, whose age ranged from 37 to 71 years.

### Cell culture

Human lung cancer cells were obtained from the Cell Bank of the Institute of Biochemistry and Cell biology, Chinese Academy of Sciences (Shanghai, China). Furthermore, all lung cancer cells were recently authenticated by short tandem repeat (Genetic Testing Biotechnology Corporation, Suzhou, China) and tested for mycoplasma contamination.

### Antibodies and reagents

The detail of all antibodies and regents were in Supplementary Information.

### Plasmids, siRNA and transfection

All siRNA were synthesized by GenePharma (Shanghai, China). The shRNAs against human PLOD2 (SHC [V2LHS_131378], SHD [V3LHS_306074]) were obtained from the shRNA and ORFeome Core at the MD Anderson Cancer Center. Scrambled shRNA (no.1864) was from Addgene (Cambridge, MA, USA). Human PGL3-basic-PLOD2-WT and the vector were obtained from the MD Anderson Cancer Center (Houston, TX, USA). The transfection was performed as described previously.^13^

### Colony formation assay, western blot, RT-PCR and immunofluorescence assay

The colony formation assays were performed in 6-well plates as described previously.^50^ After treatment for 48 h, cellular or tissue proteins were extracted and western blot was performed. The total cellular RNA was isolated with the TRIzol Reagent (Vazyme, Nanjing, China) and reverse transcribed with the HiScript QRT SuperMix for qPCR (Vazyme).The mRNA levels were measured with the SYBR Green master mix (Vazyme). The immunofluorescence assay was performed according to previous reports.^51^

### Migration and wound-healing assay

The migration assay was detected by Transwell chambers (Corning, NY, USA) and cells were allowed to migrate towards the bottom wells. After 18 h of incubation, the migrating cells were stained with DiffQuik Stain Set (Jiancheng Bioengineering Institute, Nanjing, China), photographed, and counted manually using the Image-Pro Plus Software (Rockville, MD, USA). The wound-healing assay was performed as described previously.^51^

### The exploration of transcription factors and luciferase reporter assays

First, the promoter sequence of PLOD2 was obtained via the Ensemble project. Then, the potential transcription factors were predicted by the Genomatix database.^52^ Finally, the most potential transcription factors were screened out based on the prediction score (the matrix sim). Further, luciferase activity was measured by the Dual-Luciferase Reporter Assay System (Promega, Madison, WI, USA) according to the manufacturer’s protocol.

### ChIP assay

ChIP assay was performed by Simple ChIP Plus Enzymatic Chromatin IP Kit (Cell Signaling Technology, Danvers, MA, USA) according to the manufacturer’s protocol. The final ChIP DNAs were used as templates in qPCR reactions, using primers that encompass PLOD2 promoter. The oligonucleotides of the primers were in Supplementary Information.

### Metastasis model of orthotopic implantation

Five- to six-week-old female NOD SCID mice (MARC, Nanjing University, Nanjing, China) were randomly divided into groups (six mice per group). All animals were housed under standard conditions and cared for according to protocols approved by the Experimental Animal Care Commission in China Pharmaceutical University. SCID mice were anesthetized by isoflurane inhalation, then NCI-H1975 human lung cancer cells (1 × 10^6^) were orthotopically injected through the intercostal space into the lung immediately after making a small skin incision. The 50 *μ*l cell suspension per mouse contained 25 μl Matrigel (Corning). According to previous reports,^53, 54^ after 11 days, the tumors formed and started to metastasize. After 4 weeks, the metastatic nodes were detected by gross examination and micro-PET via radioactive tracer.

### Subcutaneous xenograft tumor model

Subcutaneous xenograft tumor model was applied to evaluate the proliferation of PLOD2-knockdown cells *in vivo*. The model was performed according to our previous reports.^55^

### Immunohistochemistry

The immunohistochemistry was performed as described previously.^55^

### Statistical analysis

The relationship between P-EGFR and PLOD2 expression was analyzed via the Spearman's rank correlation test. The data were represented as the mean±S.D. of triplicate experiments performed in a parallel manner, unless otherwise indicated. The differences between means were determined via unpaired Student’s *t*-test using GraphPad Prism (La Jolla, CA, USA). The *P*-values<0.05 were considered statistically significant for all tests.

## Publisher’s Note

Springer Nature remains neutral with regard to jurisdictional claims in published maps and institutional affiliations.
