# Supplementary material for: PLOD2 regulated by transcription factor FOXA1 promotes metastasis in NSCLC
Source: Cell Death Dis. 2017 Oct 26;8(10):e3143–. doi: 10.1038/cddis.2017.553 (PMC5680920; doi:10.1038/cddis.2017.553)

**Supporting Information**

**Materials and Methods**

**Antibodies and reagents**

All the antibodies were purchased from Cell Signaling Technology, R&D Systems or Anti-EGF receptor, anti- phospho- EGF receptor (Tyr1068), anti-AKT, anti-phospho-Akt (Ser473), anti-ERK1/2, anti- phospho-ERK1/2, anti-Lamin B, β-actin and secondary antibodies were purchased from Cell Signaling Technology. Anti-PLOD2 (21214-1-AP) was purchased from Proteintech and anti- PLOD2 (MAB4445) was purchased from R&D Systems. Anti-FOXA1(#654126) was from R&D Systems and Anti-FOXA1 for ChIP(ab23738) was purchased from abcam. Goat anti-mouse IgG (H+L) Secondary antibody, Alexa Fluor® 488 conjugate and goat anti-rabbit IgG (H+L) Secondary antibody, Alexa Fluor® 594 conjugate were from Thermo Fisher Scientific. The recombinant human EGF and TGF-α was from Pepro Tech. Gefitinib, erlotinib, WZ4002, LY294002 and U0126 were purchased from Selleckchem.

**Western blotting**

The total cell lysates were extracted from the untreated/treated cells, adding phosphatase and protease inhibitors. The proteins were fractionated by 6%-15% sodium dodecyl sulfate polyacrylamide gel electrophoresis (SDS-PAGE) and electroblotted onto PVDF membrane (Millipore, USA). Then, the membranes were blocked with 5% non-fat milk and incubated with primary antibodies (dilution in 1% BSA-TBST) for 18 h at 4°C. Next, it probed with secondary antibody for 1h at room temperature. Subsequently, the expression of the target proteins was detected by Immobilon Western Chemiluminescent HRP Substrate (Millipore, USA).

**RT-PCR**

The primers used were as follows: 18S: forward primer: 5’- AGGTCTGTGATGCCC TTAGATGTC-3’and reverse primer: 5’- TCCTCGTTCATGGGGAATAATT-3’; EGFR: forward primer: 5’- AAGTGTAAGAAGTGCGAAGGGCC -3’and reverse primer: 5’-TCCAGAGGAGGAGTATGTGTGAAGG -3’; PLOD2: forward primer: 5’- GACAGCGTTCTCTTCGTCCTCA-3’and reverse primer: 5’-CTCCAGCCTT TTCGTGGTGACT-3’; FOXA1:forward primer5’-ACTCGCCTTACGGCTCTAC G-3’: and reverse primer: 5’- TGTTTAGGACGGGTCTGGAATA-3’.

**siRNA**

EGFR siRNA 1(5‘-GCAGAUCAUCAGAAAUTT-3’),

EGFR siRNA 2(5’-GGAGAUAAGUGG AGAUT-3’),

PI3KCA siRNA 1(5‘-GGACCUCAAUUCACCUCAUTT-3’),

PI3KCA siRNA 2(5‘-GCAACCUACGUGAAUGUAATT-3’),

FOXA1 siRNA A(5-‘GCGACUGGAACAGCUACUATT-3’),

FOXA1 siRNA B(5’-CCACUGGCUGUCCUUCAAUTT-3’) were synthesized by GenePharma.

**Immunohistochemistry (IHC) assay**

Breifly, the tissue sections were deparaffinized and immersed for 10 min in PBS. After antigen retrieval, the endogenous peroxidase was blocked by 3% H_2_O_2_, and non-specific staining was blocked by 5% goat serum. The sections were subsequently incubated with primary antibodies overnight at 4°C then treated with biotinylated secondary antibody for 20 minutes respectively. The sections were then exposed to DAB for 6 min. The evaluation of the IHC staining was performed by pathologist. In brief, no staining (<5%) was scored as “-”, weak staining (5-25%) was scored as “+”, moderate staining (25-65%) was scored as “++” and strong staining (>65%) was scored as “+++”.

**Chromatin immunoprecipitation (ChIP) assay**

ChIP assay was performed by SimpleChIP Plus Enzymatic Chromatin IP Kit (Cell Signaling Technology) according to the manufacturer’s protocol. The final ChIP DNAs were used as templates in qPCR reactions, using primers that encompass PLOD2 promoter. The oligonucleotides of the primers were as follows: #1, TCC CGT CGT GGT TCC TCT CT and TTC CAT GGC TTA AGA GTC TG; #2, CTG CGG AAT CGG AGC CCA A and AAA AAA GGG AAT TTT CTT AGA C; #3, CTG TTC TCA CAT TTT AAC TAG C and

ATT TAG GCA GGA GAT TCC AG.

**Supplemental Figure Legends**

**Fig. S1.** PLOD2 was an adverse prognosis marker in various tumors. (a, b) Kaplan–Meier (KM) analysis of relapse-free survival in cancer patients was from Kaplan–Meier plotter (http://kmplot.com/analysis). PLOD2 in different genders of lung cancer patients (male: n=1101, female: n=714, a), PLOD2 in lung cancer patients with a of history smoking (smoked: n=821, never smoked: n=205, b). (c, d) The mRNA expression of EGFR and PLOD2 was detected in various NSCLC cell lines. (e) The expression of P-EGFR and PLOD2 in different NSCLC cell lines. (f) The expression levels of P-EGFR and PLOD2 were consistently higher in adenocarcinoma tissues than in normal tissues of clinical samples by IHC. (g) The co-expression of P-EGFR and PLOD2 in clinical sample was analyzed by immunofluorescence assays.

**Fig. S2.** EGFR inhibitor inhibited the expression of PLOD2. (a- d) Gefinitib inhibited the expression of PLOD2 in the NCI-H1975 cell line in time- and dose- dependent manner. (e- h) Gefinitib inhibited the mRNA expression of PLOD2 in a time- and dose-dependent manner in the HCC827 cell line. The data were represented as the mean ± SD. of three independent experiments. The P-value< 0.05 were considered statistically significant for all tests.

**Fig. S3.** EGFR regulated the expression of PLOD2 and the organization of collagen. (a) WZ4002 inhibited the expression of PLOD2 in the NCI-H1975 cell line. (b) Knockdown of EGFR decreased the expression of PLOD2 by siRNA in the NCI-H1975 cell line. (c, d) Erlotinib inhibited the expression of PLOD2 in the NCI-H1975 and the HCC827 cell line. (e) Knockdown of EGFR decreased the expression of PLOD2 by siRNA in the HCC827 cell line. (f) The EGFR ligand (EGF) promoted the expression of PLOD2 in the A549 cell line. (g) The EGFR inhibitor improved the life quality of NSCLC metastasis model. The data were represented as the mean ± SD. of three independent experiments. The P-value< 0.05 were considered statistically significant for all tests.

**Fig. S4.** EGFR pathway activation could be mediated by PLOD2. (a- d) The inhibition of cell migration by Minoxidil on cancer cell line NCI-H1975 was evaluated. (e, f) The expression of PLOD2 was knocked down by shRNA in the A549 cell line. (g, h) The effects of the PLOD2 shRNA and the EGFR ligand (TGF-α) on migration were detected in A549 cell line. (i, j) The expression of PLOD2 was detected in the NCI-H1975 cell line transfected with PLOD2 plasmid. (k, l) The effects of the ectopic PLOD2 and the EGFR inhibitor (WZ4002) on migration were detected in NCI-H1975 cell line. The data were represented as the mean ± SD. of three independent experiments. The P-value< 0.05 were considered statistically significant for all tests.

**Fig. S5.** The PI3K/AKT signaling pathway promoted the expression of PLOD2 via the FOXA1 transcription factors. (a, b) Knockdown of FOXA1 decreased the expression of PLOD2 by siRNA in the HCC827 cell line. (c) The PI3K/AKT signaling pathway inhibitor (LY294002) consistently decreased the expression of FOXA1 and PLOD2 in the HCC827 cell line. (d, e) The PI3K/AKT signaling pathway inhibitor (LY294002) could inhibit the nuclear translocation by separation of the cytoplasm and nucleus and immunofluorescence assay in the HCC827 cell line. (f). 293T cells transiently co-transfected with an pIREShyg3 vector or constitutively active FOXA1 expression vector, a Renilla luciferase reporter (for normalization), and a freﬂy luciferase reporter driven by PLOD2 promoter constructs. Fireﬂy luciferase activities from a single experiment expressed as mean ± SD of triplicate wells relative to the activity of the basal reporter (pGL3), which was set at 1.0. The data were represented as the mean ± SD. of three independent experiments. The P-value< 0.05 were considered statistically significant for all tests.

**Supplemental Table 1**. The 5 transcription families were the top 5 potential transcription families from 769 matches predicted by Genomatix and JASPAR database. The matrix sim (from 0.662 to 1.00) is the prediction score of the potential transcription factors. The more close to 1, the more potential to be the transcription factor.

**Supplement table1.The potential transcription families of PLOD2 were predicted**

**from Genomatix**

| TOP 5 potential transcription factors | Detailed Family Information | Detailed Matrix Information  (Matrix sim.) | Additional Reference Evidence |
| --- | --- | --- | --- |
| 1 | Fork head domain factors | FOXA1(1.00),FOXA2(1.00),FOXP1(1.00),FOXK2(0.990),  FOXJ1(0.921),FHXA(0.884),FHXB(0.884),FOXQ1(0.876) | NO |
| 2 | NF-қb/c-rel | NF-қb(0.895),c-rel(0.971),p50(0.865),p65(0.924) | [30] |
| 3 | SMAD family | Smad3(0.997),TGFB-induced factor homeobox2-like(0.915),TIEG/EGRα(0.879) | [30] |
| 4 | STAT | STAT5(0.990),STAT(0.938),STAT5A(0.856),STAT1(0.853),  STAT6(0.851),STAT3(0.779) | [13] |
| 5 | AP | AP4(0.993),AP1(0.947),AP2(0.921) | NO |

Supplement figure.1


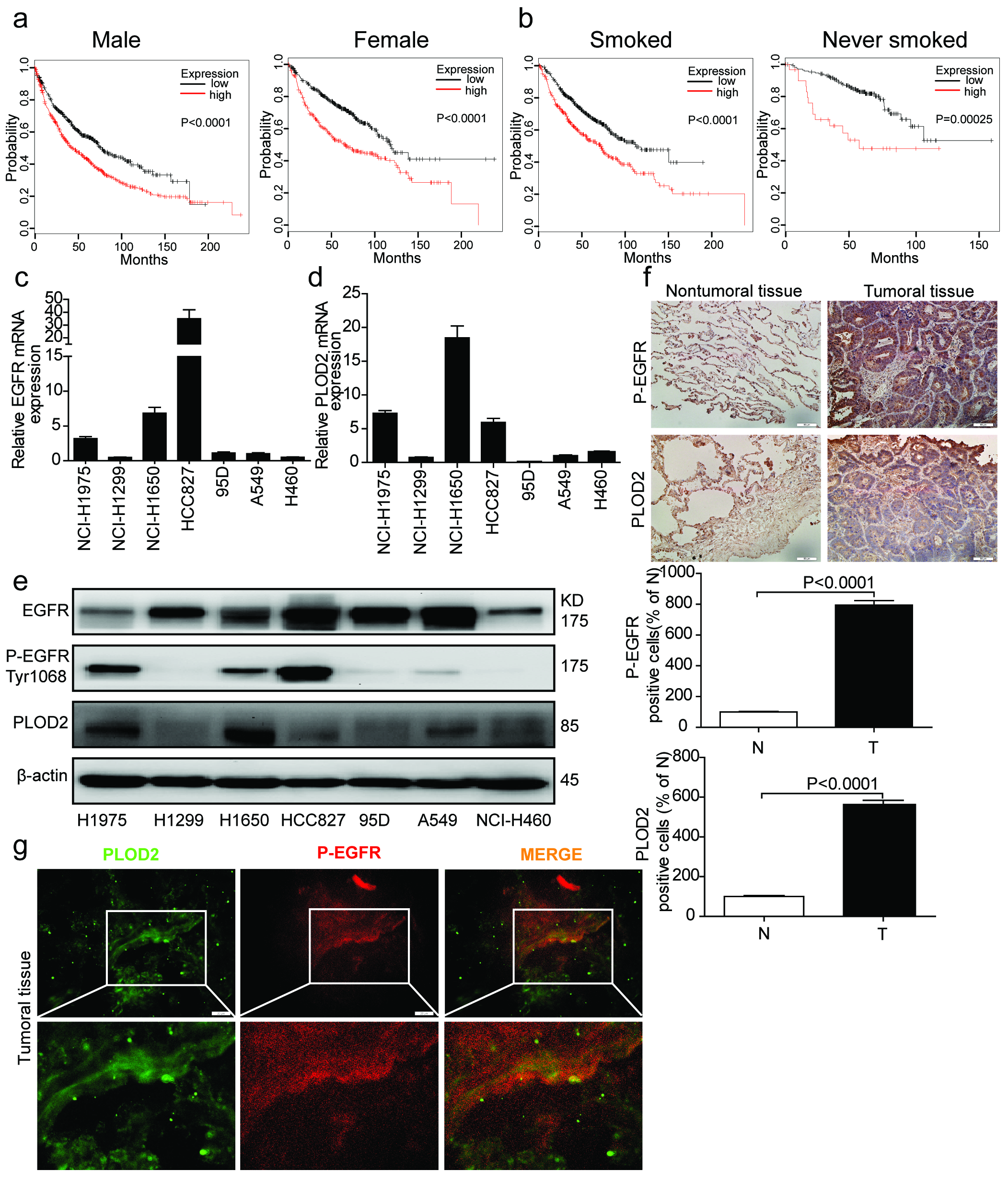


Supplement figure.2


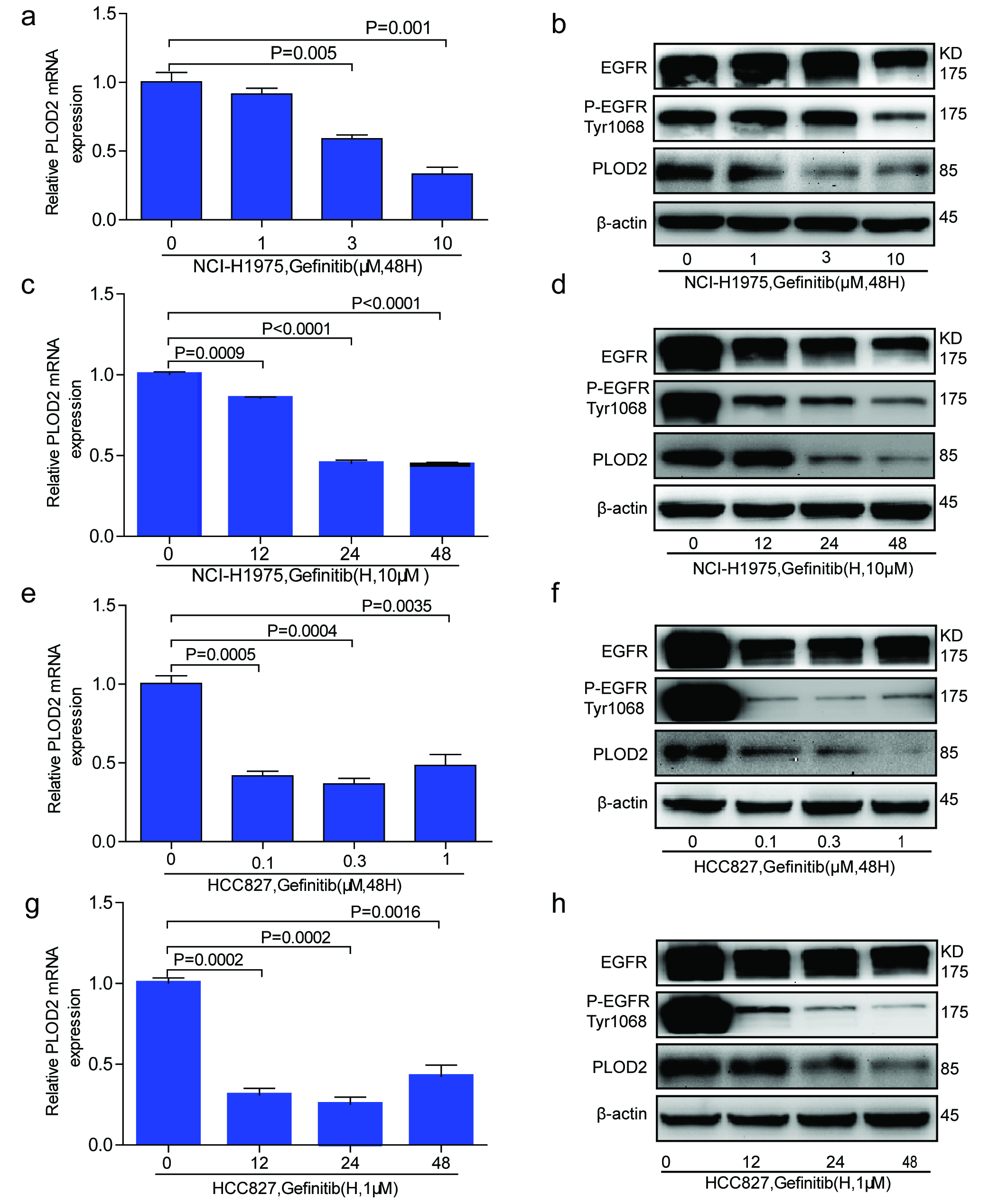


Supplement figure.3


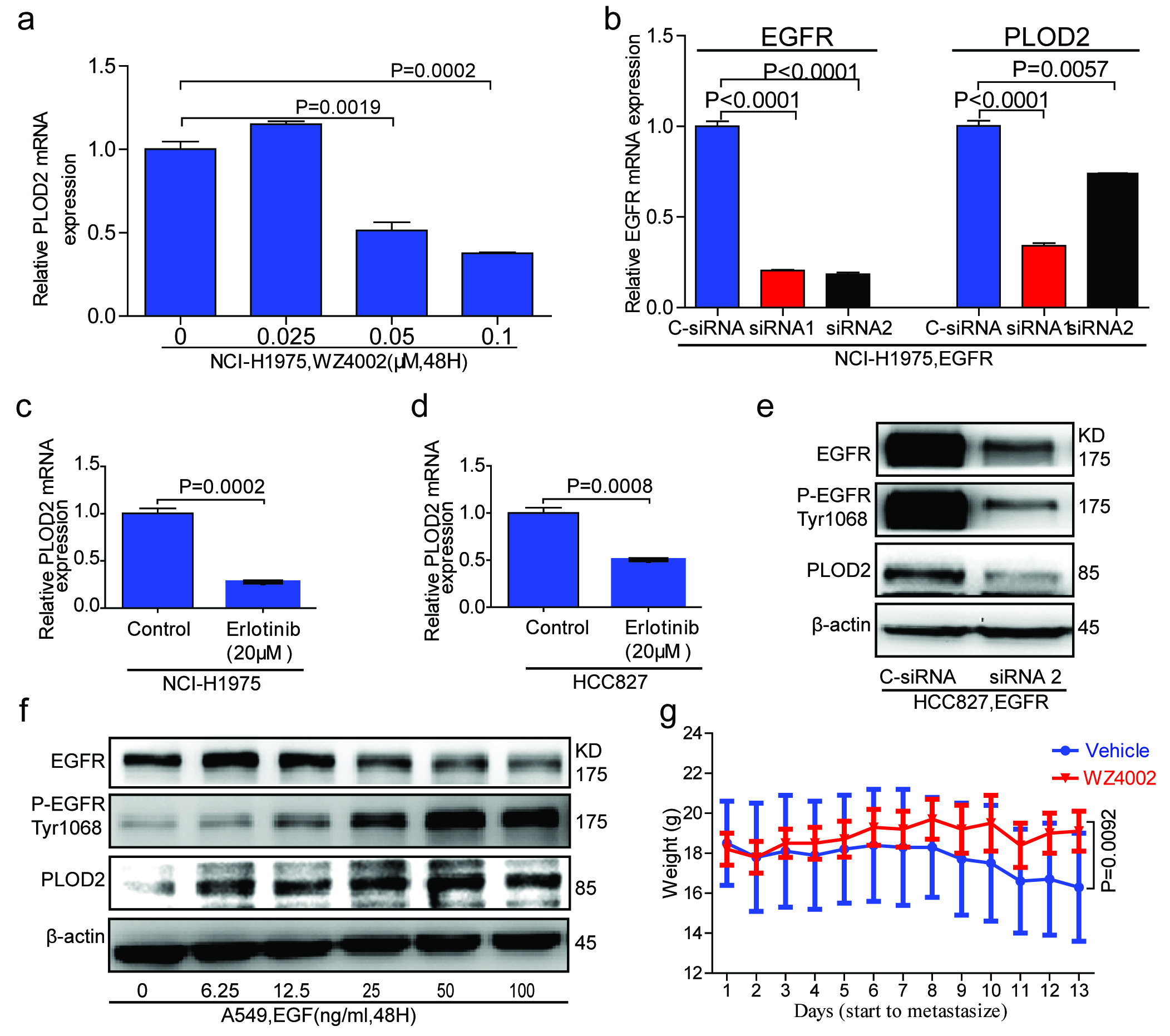


Supplement figure.4


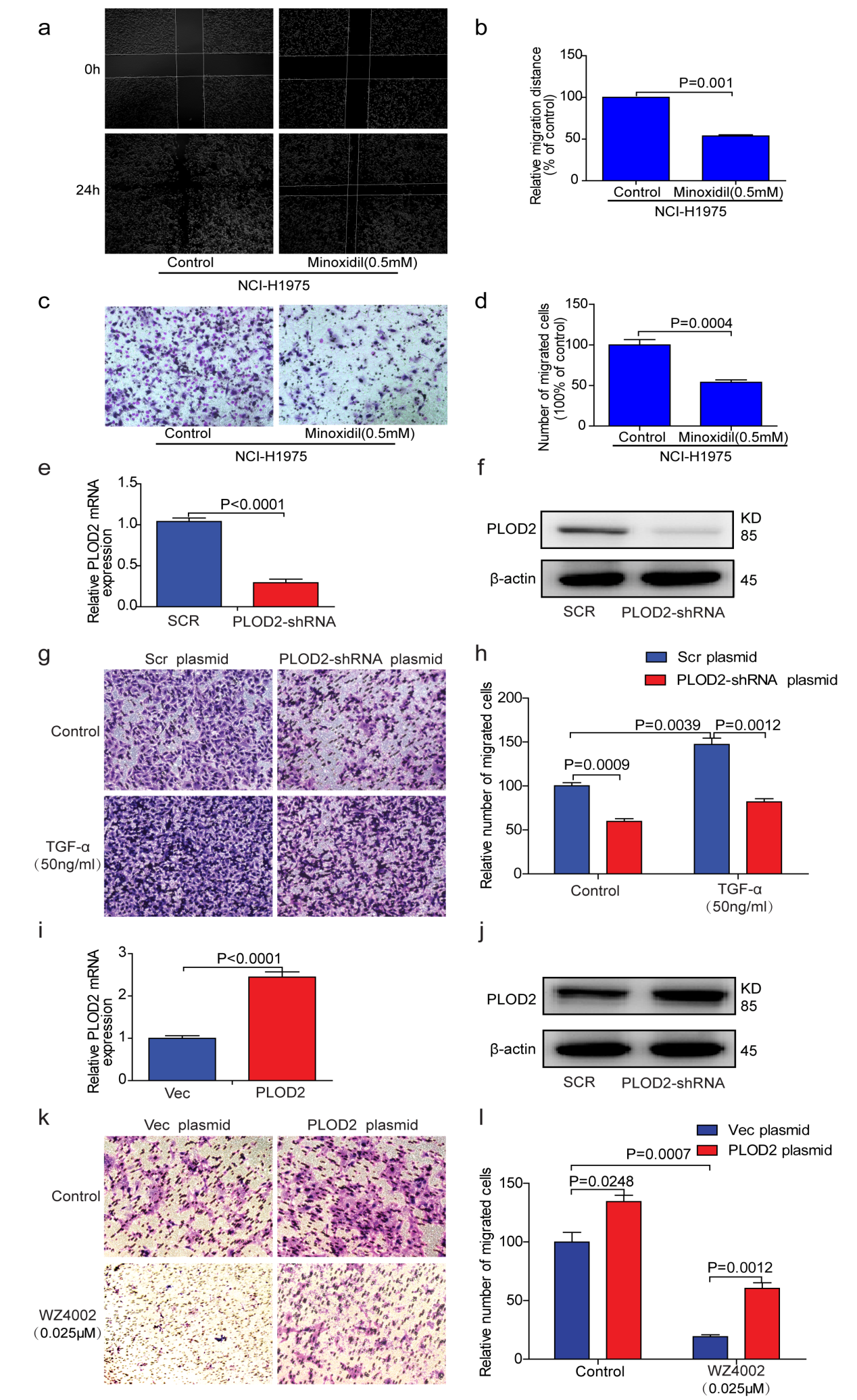


Supplement figure.5


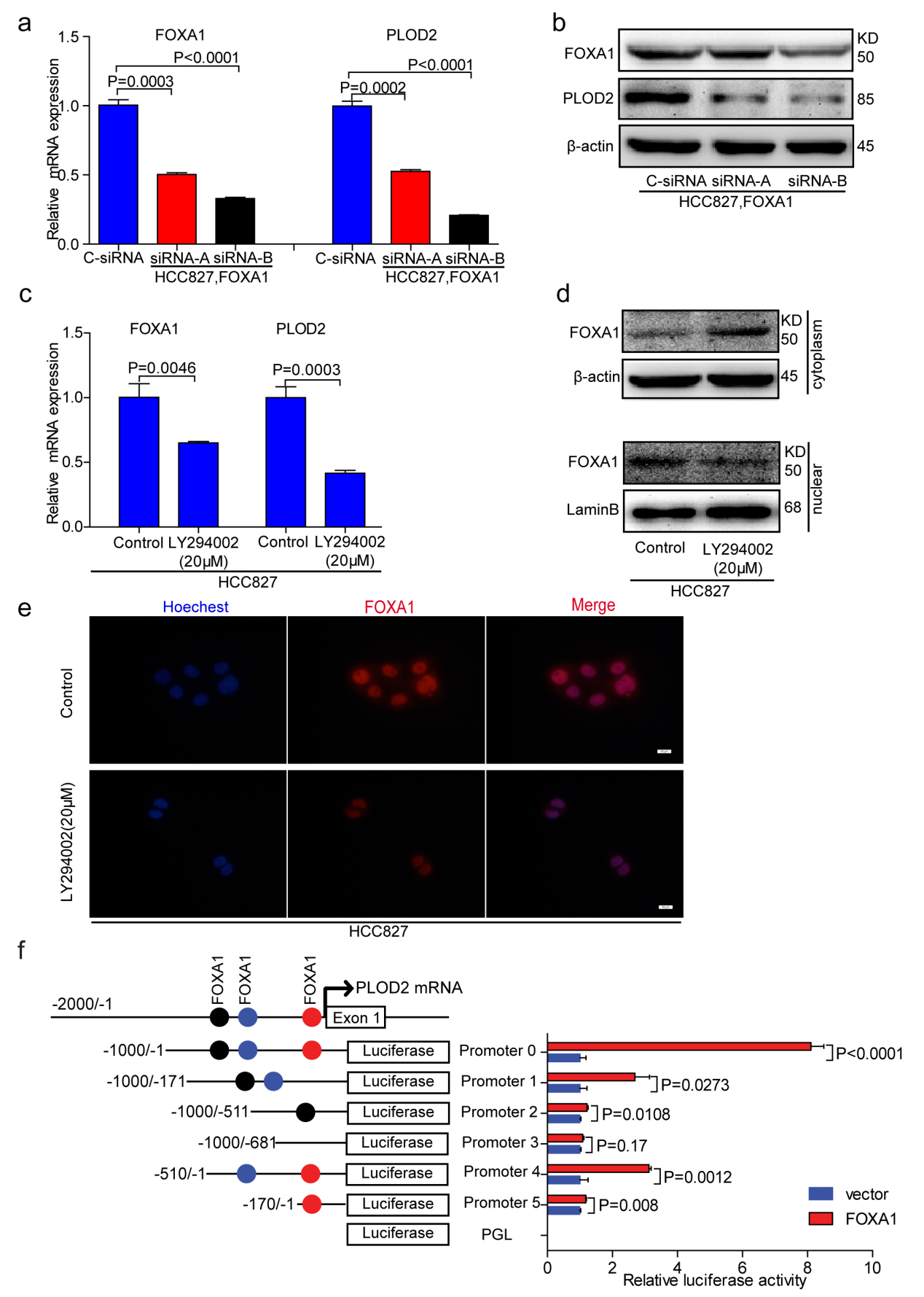

Supplement: Supplementary Information [file cddis2017553x1.docx]
